# Shared neuroimmune and oxidative pathways underpinning Chagas disease and major depressive disorder

**DOI:** 10.1038/s41398-020-01105-9

**Published:** 2020-12-02

**Authors:** Eduardo Duarte-Silva, Michael Maes, Danielle Macedo, Wilson Savino, Christina Alves Peixoto

**Affiliations:** 1 Laboratory of Ultrastructure. Aggeu Magalhães Institute, Oswaldo Cruz Foundation (FIOCRUZ), Recife, Brazil; 2 Postgraduate Program in Biosciences and Biotechnology for Health (PPGBBS), Aggeu Magalhães Institute, Oswaldo Cruz Foundation, Recife, Brazil; 3 Network of Immunity in Infection, Malignancy and Autoimmunity (NIIMA), Universal Scientific Education and Research Network (USERN), Recife, Brazil; 4 Department of Psychiatry, Faculty of Medicine, Chulalongkorn University, Bangkok, Thailand; 5 Neuropsychopharmacology Laboratory, Drug Research and Development Center, Faculty of Medicine, Federal University of Ceará, Fortaleza, Brazil; 6 Department of Physiology and Pharmacology, Faculty of Medicine, Federal University of Ceará, Fortaleza, Brazil; 7 National Institute of Science and Technology on Translational Medicine (INCT-TM, CNPq), Ribeirão Preto, Brazil; 8 Laboratory on Thymus Research, Oswaldo Cruz Institute, Oswaldo Cruz Foundation, Rio de Janeiro, Brazil; 9 National Institute of Science and Technology on Neuroimmunomodulation (INCT-NIM), Oswaldo Cruz Institute, Oswaldo Cruz Foundation, Rio de Janeiro, Brazil; 10 Rio de Janeiro Research Network on Neuroinflammation, Oswaldo Cruz Institute, Oswaldo Cruz Foundation, Rio de Janeiro, Brazil

**Keywords:** Molecular neuroscience, Depression

## Abstract

The cellular and molecular basis to understand the relationship between Chagas disease (CD), a infection caused by *Trypanosoma cruzi*, and depression, a common psychiatric comorbidity in CD patients, is largely unknown. Clinical studies show an association between CD and depression and preclinical evidence suggests that depressive-like behaviors in *T. cruzi* infected mice are due, at least partially, to immune dysregulation. However, mechanistic studies regarding this issue are still lacking. Herein, we present and discuss the state of art of data on CD and depression, and revise the mechanisms that may explain the development of depression in CD. We also discuss how the knowledge generated by current and future data may contribute to the discovery of new mechanisms underlying depressive symptoms associated with CD and, hence, to the identification of new therapeutic targets, which ultimately may change the way we see and treat CD and its psychiatric comorbidities.

## Introduction

One hundred and eleven years after its discovery by Carlos Chagas, American trypanosomiasis, also known as Chagas disease (CD), continues to be a neglected disease, although it affects more than eight million people worldwide, especially in Latin America, where the disease is endemic^1,2^. However, due to human migration from endemic to other non-endemic areas, such as United States, Canada, Europe, Japan and Australia, CD is now considered a concern in these places^3^. Regardless of the place, CD causes a high socioeconomic burden^4^, which may be significantly reduced when approaches to control disease are used, such as vector control, screening strategies and treating the disease at an early stage^2,3^.

Chagas Disease is caused by the protozoan *Trypanosoma cruzi* and yields inflammation predominantly in the heart and gastrointestinal tract (GIT). Since the parasite also has tropism to brain cells such as microglia, astrocytes and neurons (at least in vitro)^5^ it can also affect the nervous system^6^.

In non-endemic areas, CD may be transmitted mainly via blood transfusion, organ donation, congenital transmission during pregnancy, and oral route when food and drinks contaminated with feces of triatomine bugs are ingested^7–9^. In endemic areas, CD is transmitted mainly via vectors, insects known as triatomines which acquire the parasite after a blood meal of infected humans or other animals^7^. After infection with *T. cruzi* the disease follows an acute and a chronic phase. The latter is characterized by intense parasitemia, but is usually asymptomatic or the patients can have a few symptoms, such as fever, inflammation, lymphadenopathy, tachycardia, hepatosplenomegaly, fatigue, and rarely myocarditis and meningoencephalitis^10,11^. These symptoms spontaneously disappear in most patients^1,7^. On the other hand, one third of the patients progress to the chronic phase of disease. Chronic CD usually starts with a *latency period* called chronic indeterminate form which can last for more than 30 years or throughout life, remaining unnoticed. The majority of the infected individuals remain as such. Nevertheless, in some cases, this indeterminate form is followed by symptomatic forms of the chronic phase, characterized by decrease in parasitemia and by cardiac, digestive or neurological manifestations^1,11^. Moreover, some preclinical and clinical studies reported that, in the chronic phase of CD, there are behavioral changes and the occurrence of psychiatric comorbidities such as anxiety and depression, conditions that, in principle, cannot solely be explained by the psychological *status* of the patient^12–18^.

Major depressive disorder (MDD) is a psychiatric disorder that affects sleep, eating behaviors, cognition, emotions, and energy balance, causing feelings of guilt, impaired cognitive functions, mood changes, motor dysfunctions and suicidal thoughts, which affect millions of people worldwide. Besides predisposing to other chronic diseases, such as obesity, heart disease and diabetes, depression is also responsible for deaths caused by suicide^19^. MDD is now conceptualized as an immune-inflammatory disorder whereby activated neuroimmune and related oxidative pathways induce changes in brain neurons leading to the symptoms of depression^20,21^. This psychiatric disorder may worsen the course of CD and add to the intense burden in those patients, which ultimately have their life quality severely compromised^18^.

Therefore, the aims of this review article are: (i) to review the preclinical and clinical data showing the occurrence of depression in CD, providing new insights into their relationship; (ii) to review the shared pathways that underpin Chagas disease and comorbid depression; and (iii) to discuss how the understanding of the neuropsychiatric aspect of CD is key to an in-depth view of its pathogenesis and to develop novel approaches to treat it.

## Search strategy and selection criteria

We used Pubmed (MEDLINE) as the database to collect the studies available in this review. The search strategy comprised the use of following terms, alone or in combination: “major depressive disorder” [MeSH], “depression” [MeSH], “Chagas disease” [MeSH], “Trypanosoma cruzi” [MeSH], “anxiety” [MeSH], “oxidative stress” [MeSH], “antioxidant” [MeSH], “cytokines” [MeSH], “mitochondrial dysfunction” [MeSH], “autoimmunity” [MeSH], “cortisol” [MeSH], “hypoactivity” [MeSH], “HPA axis” [MeSH]. Articles resulting from these searches and relevant references cited in those articles were reviewed. We selected the papers written in English, Spanish and Portuguese language, and considered papers regardless of the year of publication.

## Evidence from animal and human studies linking Chagas disease and depression: what do we know?

Pre-clinical and clinical studies show that depressive- and anxiety-like behaviors are common in Chagas disease (Table 1), which essentially differs from sickness behavior, a natural physiological response triggered by tissue trauma or infection and coordinated by the immune system via peripheral release of cytokines, such as IL-1β and TNF-α, which aims at restoring homeostasis by adjusting the body’s priorities to eradicate the pathogen. The body temperature is increased, causing fever, and the animals experience anhedonia, social withdrawal, decreased locomotor activity, to mention just a few^22,23^. Sickness behavior has been recognized as a mechanism responsible for behavioral alterations in some animal models of disease, such as Experimental Autoimmune Encephalomyelitis (EAE), a mouse model of Multiple Sclerosis (MS)^24^, but it does not seem to be the case in experimental CD. In this regard, acute or chronic infection of C57BL/6—a mice strain resistant to develop *T. cruzi*-induced neuroinflammation—and C3H/He—susceptible to develop meningoencephalitis—with the C*olombian T. cruzi* strain—which has tropism for heart and central nervous system (CNS)—did not result in fever, weight loss and apathy, classical signs of sickness behavior in mice^14^. Therefore, behavioral changes observed in *T.cruzi*-infected mice are likely a result of underlying depressive-like symptoms. Accordingly, when infected mice were subjected to tail suspension test (TST) and forced swim test (FST) (tests commonly used to evaluate depressive-like behaviors in mice)^25,26^, they exhibited increased immobility time in the FST, corroborating the presence of depressive-like behaviors in both acute and chronic phases of the disease and showing that these behavioral changes were not due to acute CNS inflammation^14^. These results were corroborated in another study with C57BL/6 mice^15^, which also showed that infected chronically mice had anxiety-like behavior as assessed by a reduced number of entries and time in the open arms of the elevated plus maze test (EPMT, a test usually taken to evaluate anxiety-like behavior in mice)^27^. In humans, anxiety is also a prominent feature of Chagas disease^12,13,16^.

**Table 1:** Comparative table showing pre-clinical and clinical data and main findings on CD and depression.

Clinical studies	Main findings
Jörg et al.1981	Depression in 81·1% of patients and neurocognitive disturbances (confusion, weakness of muscular-tendineous reflexes, speech disturbance, delirant ideias) in CD patients
Mangone et al. 1994	CD patients have cognitive impairment
Marchi and Gurgel. 1998	Depression in CD patients
Hueb et al. 2005	CD patients have cognitive and mood dysfunctions psychological changes (Review)
Marchi and Gurgel. 2011	Depression in CD patients: two patients (3·3%) maintained the same mild depressive state as observed 13 years earlier; one participant (1·7%) maintained his clinical condition at a moderate level; five patients (8·3%) improved from moderate to mild depression, and two (3·3%) showed remission from their depressive episode. Only one (1·7%) patient progressed from mild to moderate depression
Ozaki et al. 2011	Mild type of depression in CD patients
Jackson et al. 2012	Depression in 28·5% and anxiety in 58·4% of migrating CD patients
Suman et al. 2017	Depression in CD patients
**Pre-clinical studies**	**Main findings**
Vilar-Pereira et al. 2012	In acute and chronic phase: absence of locomotor/exploratory activity in CH3/He mice and presence in C57BL/6 mice; absence of sickness behavior; depressive-like behavior; Increased IDO mRNA expression
Vilar-Pereira et al. 2015	Depressive-like behavior; anxiety-like behavior; decreased locomotor/exploratory behavior; decreased motor coordination; Absence of loss of muscle strenght and sickness behavior in C57BL/6 during the chronic phase

In rats, *T. cruzi* infection triggered sleep disturbances and memory deficits^28^. This is an important flaw that must be addressed in future studies because sleep disturbances and memory impairments are common symptoms of depression^29^. Furthermore, besides depression, neurocognitive disturbances^30^ and cognitive deficits^31^ are also reported in chagasic patients, who may also suffer from fear^16^.

Chagas disease has a strong psychological component driving its progression and the progression of its comorbidities, such as depression and anxiety. For instance, a previous study revealed that the fact of being diagnosed with CD could trigger psychological changes and cause mood disturbances^16^. Although preclinical studies support the notion that psychological factors are not the *only* causative agents of depression in Chagas disease, since it may be caused by other biological mechanisms (discussed below in section 4), one should not disregard the influence of psychological factors in the disease course, as currently, accumulated evidences suggest a bidirectional communication between the brain and other physiological systems, such as immune and endocrine systems. In this regard, psychological factors and thoughts affect brain and immunity and vice-versa, ultimately influencing disease acquisition, resilience and progression^32,33^.

Chagas disease is not only related to the emergency of physical symptoms, but also causes neurocognitive, psychological disturbances and mood changes. For instance, previous studies identified the presence of depression in adults and children with CD most often using Hamilton Depression Rating Scale (HAM-D), Montgomery-Asberg Depression Rating Scale (MADRS) and Beck Depression Inventory (BDI)^12,13,16–18,30,34^. It is noteworthy to mention that depressive symptoms in chagasic patients occur with varying degrees of severity. However, the mild subtype of depression was observed most frequently^17,18^. Interestingly, depressive symptoms seem to vary according to disease form, since higher BDI scores were observed in patients with the digestive form of disease, followed by the cardiac form and by the indeterminate form^18^.

In sum, pre-clinical studies show that Chagas disease induced by type I *T. cruzi* is accompanied by depressive and anxiety-like behaviors in the chronic phase of disease, which are not a result of sickness behavior or acute inflammation. On the other hand, clinical studies show that Chagas disease is accompanied by psychological and mood disturbances, specifically the mild form of major depressive disorder, and that the status of the patients may be influenced by psychosocial factors.

## Possible mechanisms linking depression with Chagas disease: How this bidirectional interaction determines the patient’s state and fate?

### Tryptophan metabolism and *T. cruzi*: a dangerous dyad behind depressive symptoms

Tryptophan is an essential amino acid relevant to physiological processes, such as regulation of CNS^35^ and immune functions^36–38^. Tryptophan has two distinct metabolic pathways, namely the serotonin (5-HT) pathway and the oxidative or TRYCATs pathway^39^, which leads to the production of the neurotransmitter 5-HT/melatonin and tryphophan catabolites and nicotinamide/NAD^+^, respectively. The TRYCATs’ metabolites exert neuroprotective and detrimental effects, such as kynurenic acid (KYNA) and quinolinic acid (QUINA)/3-hydroxikynurenin (3-HK), respectively^40,41^. Two enzymes are necessary for the conversion of tryptophan in the TRYCATs pathway: tryptophan 2,3-dioxygenase (TDO) and indoleamine 2,3-dioxygenase (IDO). The activity of the latter is increased in a pro-inflammatory milieu^42,43^. Interestingly, increased levels of IDO as well as alteration in tryptophan metabolism were detected in the brains of animals exposed to models of depression^44^. Consequently, this pathway is implicated in the pathophysiology of depression^40,45^. It is important to note that local and transient tryptophan depletion limits pathogen growth by inhibiting pathogen metabolism due to lack of substrates^46,47^. However, prolonged tryptophan depletion has detrimental implications for immunity and behavior.

Increased IDO activity is associated with resistance to acute *T.cruzi* infection and was critical in the control of the parasite’s replication in macrophages^48^. Conversely, the administration of a pharmacological inhibitor of IDO1, 1-methyl-d-tryptophan (1-MT), to infected mice increased their susceptibility to infection, parasite’s number and exacerbated infection-associated pathology, such as the presence of numerous inflammatory foci in the heart and skeletal muscle^48^. Moreover, 3-HK and 3-hydroxyanthranilic acid (3-HAA) inhibited intracellular *T. cruzi* replication^48^. Interestingly, it was also observed that increased IDO mRNA expression in the CNS in acute and chronic *T. cruzi* infection paralleled depressive-like behavioral alterations infected mice^14^, suggesting that depletion of tryptophan affects the biosynthesis of monoamines, such as 5-HT.

Overall, it seems that both mechanisms underpinning depressive-like behaviors in Chagas disease and depression are associated with IDO activation and, consequently, with the synthesis of neurotoxic TRYCATs including 3HK and QUINA. The latter are associated with the onset of depression and with interferon (IFN)-α-induced major depression, since immunotherapy of chronic hepatitis C patients with IFN-α is associated with activation of T cells, IDO activity and TRYCATs formation, as well as increased serum levels of IL-6 and IL-8^40,49^.

An important issue that deserves attention is that inhibition of IDO activity may not work as a therapeutic strategy in Chagas disease and comorbid depression, since inhibition of IDO would also block its beneficial effects. In this regard, mechanisms other than IDO inhibition, such as inhibition of TNF-α signaling, can be trialed.

### Heart dysfunction, Chagas Disease and depression

Epidemiological observations indicate that Cardiovascular Disease (CVD) and depression are highly comorbid with multiple bidirectional relationships whereby depression increases its risk and vice-versa^50–52^. There are multiple pathways that may explain this comorbidity, including activated immune-inflammatory pathways (including TNF-α and IL-6 driven mechanisms), neurotoxic TRYCATs, lipid peroxidation and lowered levels of antioxidants, which are associated with major depression^53^.

A recent study suggests that the comorbidity between depression and CVD, specifically in the case of Coronary Heart Disease, relies on environmental factors, such as IL-6, C-reactive protein (CRP) and triglycerides^54^. The development of CVD is the result of immune activation with accompanying oxidative stress leading to autoimmune responses to oxidized low-density lipoproteins (ox-LDL) and the same mechanisms are also related to the pathophysiology of major depression^53,55^.

In this respect, a hypothesized was raised, suggesting that depression and CVD are outcomes of a same underlying process^51^. Accordingly, the activation of innate immunity and the subsequent inflammation triggered by *T. cruzi* would lead to atherosclerosis and changes in vascular dynamics. The same pathways also underpin the comorbidity between depression and CVD^53^. As such, the neuro-immune pathways in Chagas disease (including pro-inflammatory cytokines and increased neurotoxic TRYCATs) might drive depression.

### Activation of immune-inflammatory pathways in depression and Chagas disease

Chagas disease is characterized by activation of cell mediated immunity (CMI), including activation of Th-1, Th-17 cells and T CD8^+^ lymphocytes as well as the secretion of pro-inflammatory cytokines (PICs), such as TNF-α, IFN-γ, IL-6, IL-17^56–59^. Although this is essential to parasite elimination, prolonged exposure to such PICs may cause tissue damage, especially in the heart, leading to fibrosis^58^. In a second vein, it is worthy to mention that peripheral cytokines may enter the CNS via Th-17-induced blood-brain barrier (BBB) dysfunction^60^ and induce neuroinflammation, mood and behavioral alterations.

In mice, increased plasma TNF-α levels were observed that after experimental *T. cruzi* infection^14^. Interestingly, TNF-α modulators (anti-TNF and pentoxifylline [PTX], a vasodilator), as well as the antidepressant fluoxetine, reduced depressive-like alterations in infected mice^14^, which suggests a possible role for systemically produced TNF-α in *T. cruzi*-induced depression. In this respect, it is important to note that depression is accompanied by increased levels of PICs, such as TNF-α^61,62^. Furthermore, depression is also characterized by activation of CMI, inflammation and increased levels of acute phase proteins (APPs)^35,63^. Interestingly, TNF-α was also shown to facilitate invasion of astrocytes by *T. cruzi* in vitro and this was accompanied by a shift in astrocytes profile, which became pro-inflammatory and secreted TNF-α and IL-6^64^. This may lead to neuroinflammation and microglia activation, which may further affect neurotransmission and mood. The specific effect of TNF-α upon astrocyte invasion by *T. cruzi* was blocked by pentoxylin and Infliximab - a TNF neutralizing antibody^64,65^. Of note, another PIC, namely, IFN-γ also facilitated astrocyte invasion by the parasite in vitro^65^. All in all, during *T. cruzi* infection and depression activation of immune-inflammatory pathways are observed, which results in peripheral and CNS inflammation and mood changes.

### Activation of Oxidative & Nitrosative Stress (O&NS) pathways and reduced antioxidant capacity in depression and Chagas disease

Depression is usually followed by activation of Oxidative & Nitrosative Stress (O&NS) pathways. As a result, depressed subjects have increased levels of O&NS markers, such as malondialdehyde (MDA), 8-hydroxy2-deoxyguanosine (8-OHdG), DNA, RNA and mitochondrial DNA (mtDNA) damage, telomere shortening, as well as oxidized damage-associated molecular patterns (ox-DAMPS), comprising oxidized low-density lipoprotein and, oxidized phospholipids (ox-PLP)^66^. Moreover, high expression of reactive oxygen species (ROS) and reactive nitrogen species (RNS), as well as increased expression of inducible nitric oxide synthase (iNOS) are also reported^66^. Interestingly, O&NS pathways can be activated by immune-inflammatory pathways and vice-versa. However, in Chagas disease O&NS pathways are primarily driven by parasite-induced (neuro)inflammation. Moreover, excessive O&NS pathways can be a result of lowered antioxidant capacity or dysfunctional redox status^66^.

In the context of Chagas disease, it should be pointed out that O&NS pathways play a key role in *T. cruzi* infection^67–70^. For example, high iNOS expression, high levels of H_2_O_2_, 4-hydroxynoneal (4-HNE) - a lipid peroxidation marker- as well as 3-nitrotyrosin (3-NT)—a nitrosative stress marker -, advanced oxidation protein products (AOPPs) - markers of inflammation and oxidative stress - were detected in the heart after infection with *T. cruzi*^67,68^, which demonstrates that the parasite induces a strong oxidative and nitrosative damage to cardiomyocytes. Furthermore, increased oxidative stress is also reported in the brain of acutely infected mice, as demonstrated by high MDA levels^71^. Moreover, high levels of AOPPs, nitrite, lipid peroxides, MDA and 3-NT were detected in the serum of chagasic patients^72,73^, as well as high myocardial mitochondrial MDA levels^74^.

Studies have shown that depressed individuals have lowered levels of antioxidants and antioxidant enzymes, such as vitamin C, vitamin E, gluthatione, gluthatione peroxidase (GPX), superoxide dismutase (SOD), tryptophan, 5-HT, Zinc, coenzyme Q10^35^. As such, neutralization and counterbalance of O&NS pathways-induced cell/tissue damage is not achieved, which plays a key role in disease progression. Accordingly, CD is also characterized by reduced antioxidant capacity, lowered vitamins E and C and nuclear factor erythroid 2-related factor (nrf2) levels^67,68^. Moreover, decreased levels of GPX and SOD were detected in chagasic patients when compared to control subjects^72,75^ as well as reduced glutathione^74^. Interestingly, in chronic chagasic patients, the serum concentration of antioxidants vary depending on disease form: subjects with the indeterminate form display higher GPX and SOD levels in comparison to patients with the symptomatic form of disease^76^. All in all, as a shared mechanism, reduced antioxidant capacity may underlie depressive symptoms in Chagas disease and depression.

### Gut microbiota and inflammation in depression and Chagas disease

New evidence has pointed to the role of gut microbiota in mood, neurodevelopmental and neurodegenerative disorders. It is now becoming accepted that gut microbiota dysfunction or *dysbiosis* is associated with depression. Yet, the precise mechanisms underpinning this pathway are still being investigated^77,78^. In humans, increased levels of immunoglobulin M (IgM) and A (IgA) against gut microbiota-derived lipopolysaccharide (LPS) were found, which is a result of bacterial or LPS translocation from the gut to mesenteric lymph nodes (MLNs) or bloodstream due to a loosened intestinal barrier^79,80^. Once outside the gut lumen, LPS binds to toll-like receptors 2 (TLR2) or 4 (TLR4), triggering the activation of immune-inflammatory pathways, consequently reinforcing inflammation and O&NS^66^. Not surprisingly, gut microbiota dysfunction has also been reported in animal model of Chagas disease and in chagasic patients^81,82^, raising the possibility that depression in CD may also be caused by gut microbiota changes.

### Mitochondrial dysfunction in depression and Chagas disease

Data have shown that lowered ATP production and dysfunction in the mitochondrial respiratory chain complexes as well as mtDNA damage are important features of depression^66^. Mitochondrial dysfunction has now been linked to a plethora of diseases, including, but not limited to neuropsychiatric disorders, such as depression^83^. In a second vein, *T. cruzi* infection also affects mitochondrial function. For example, lowered ATP production^84^, deficits in respiratory chain complexes (I-IV)^84,85^ as well as reductions in mtDNA content and lowered mRNA encoding for respiratory chain complexes^86^, oxidation of respiratory complexes^87^ are observed in preclinical studies. In humans, increased oxidative stress, mtDNA damage and consequent mtDNA loss were observed in the heart tissue as well as lowered expression of genes of the respiratory chain, reduced ATP synthesis and activities of the respiratory chain complexes^74,88,89^. Nevertheless, whether these changes also occur in the brain and affect mood and behavior still demands further research. To summarize, it is conceivable that mitochondrial dysfunction is a shared mechanism in Chagas disease and depression and may underlie depressive symptoms.

### Autoimmunity in depression and Chagas disease

Due to increased inflammatory and oxidative and nitrosative stress (IO&NS) pathways observed not only in depression, but also in Chagas disease, ROS/RNS can react with nucleic acids, aminoacids, phospholipids, and proteins and generate neoepitopes against which an (auto)immune response can be mounted. As a consequence, cell/tissue damage is further spread^90^. Depression is also often associated with autoimmunity^91–93^. Interestingly, cardiac and CNS autoimmunity has also been reported in Chagas disease^94–98^. It is plausible to speculate that, due to increased IO&NS pathways seen in Chagas disease, neoepitopes may also be formed, leading to changes relevant to the explanation of how the observed mood alterations arise in chagasic subjects.

### The Compensatory Immune-Regulatory Reflex System (CIRS) in depression

Sepsis, tissue damage and trauma are followed by an acute inflammatory reaction aiming to eliminate the triggering factors. This condition is termed systemic inflammatory response syndrome (SIRS) and it comprises cell-mediated and humoral immune activation, such as macrophage activation, Th-1 and Th-17-mediated responses and secretion of PICs. SIRS is usually accompanied by the compensatory anti-inflammatory response syndrome (CARS), which attenuates SIRS and reestablish immune homeostasis. As such, it is characterized by immunosuppressive cells and molecules, such as Th-2- and T regulatory (Treg)- mediated immune responses and the secretion of IL-10, TGF-β, IL-4, soluble IL-1 receptor antagonist (sIL-1RA), soluble TNF-α receptor (sTNF-R) and T cell anergy. A process similar but less severe to CARS is proposed to occur in depression and therefore it was named CIRS. Besides the aforementioned mechanisms, CIRS is also characterized by increased levels of acute phase proteins (APPs) and activation of IDO, which also have dampening effects on immunity^22,99^.

### Immunoneuroendocrine changes in Chagas disease and depression

A number of studies revealed *immunoneuroendocrine* disturbances in *T. cruzi* infection^5,100^. For example, hypothalamus-pituitary-adrenal (HPA) axis dysfunction is observed after acute infection with the parasite. Infected animals displayed elevated corticosterone levels, lowered amount of corticotropin-releasing hormone (CRH) and similar quantities of adrenocorticotropic hormone (ACTH) in the serum and in the hypothalamus when compared to control animals^101^. In chronic chagasic patients, reduced levels of the adrenal androgen dehydroepiandrosterone sulfate (DHEAs) were observed in all disease forms (“indeterminate”, “mild to moderate” and “severe” cardiac)^102^. Furthermore, decreased levels of plasma cortisol (hypocortisolemia) in comparison to healthy control subjects were also reported in chagasic patients^103^. Reduced levels of DHEA and increased cortisol/DHEA ratio were documented in MDD patients^104,105^, despite apparently contradicting findings^106^. In depression, high levels of cortisol (hypercortisolemia) and CRH due to HPA axis hyperactivity and resistance to GC are usually found^107–110^, despite opposing findings^111,112^. Activation of the HPA axis during infection may have a protective role by limiting the development of an overzealous immune response. As such, it is expected that glucocorticoid and DHEA levels rise, exerting immunosuppressive effects. At a first glance, the aforementioned results of DHEA and cortisol in chronic chagasic patients may indicate that the inflammatory response in these patients is not being properly controlled, ensuring disease progression, cell/tissue damage and autoimmunity^100^. Taken together, in the context of Chagas disease and considering the HPA axis hypoactivity and lowered cortisol levels, depressive symptoms in chagasic patients may probably arise as (i) a result of uncontrolled immune-inflammatory pathways rather than HPA axis hyperactivity and hypercortisolemia; (ii) reduced amount of DHEA, which is a neurosteroid with antidepressant activity^113^, although these results should be interpreted with caution.

## Concluding remarks and future perspectives

Depression is a common psychiatric comorbidity in Chagas disease and occurs in varying degrees of severity. As a consequence, it compromises the quality of life of chagasic patients. Interestingly, both Chagas disease and depression are often underdiagnosed. Therefore, the possibility of treating Chagas disease and comorbid depression at an early stage is very unlikely and demands more efficacious screening and disease control. However, this can be achieved by combining the knowledge of neuropsychiatry when tackling Chagas disease.

**Fig. 1:**
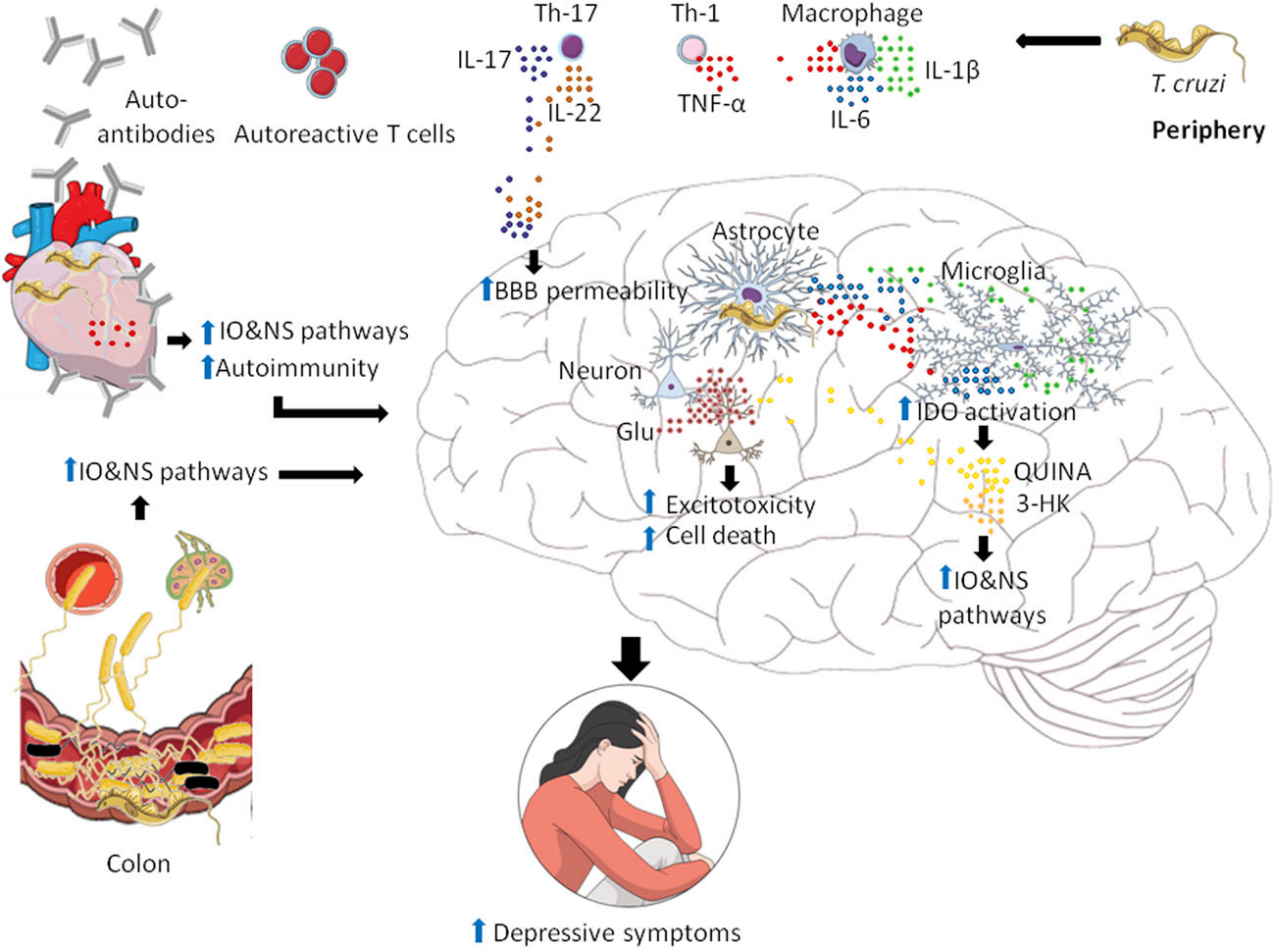
Schematic summarizing the shared inflammatory and oxidative and nitrosative stress (IO&NS) pathways underlying depressive symptoms in Chagas disease and depression. After infection with *T. cruzi* in peripheral sites, activation of (neuro)immune-inflammatory pathways occur. As a result, activation of cell-mediated immunity (CMI) resulting in activation of marophages, Th-1 and Th-17-mediated immune responses with consequent secretion of pro-inflammatory cytokines (PICs), such as TNF-α, IL-6, IL-1β, IL-17, IL-22, and activation of indoleamine-2,3-dioxygenase (IDO) with production of TRYCATs catabolites, such as quinolinic acid (QUINA) and 3-hydroxykynurenin (3-HK), take place. Moreover, decreased levels of dehydroepiandrosterone (DHEA) are also observed (not shown). As a consequence of immune-inflammatory pathways activation, oxidative and nitrosative stress (O&NS) pathways are also activated, le2ading to increased production of reactive oxygen species (ROS) and reactive nitrogen species (RNS), high levels of inducible nitric oxide synthase (iNOS) (not shown). As a consequence, this may leads to the formation of neopitopes and increased autoimmunity as well as mitochondrial dysfunction and reduced ATP production (not shown). The parasite can reach other body sites via the bloodstream (not shown), such as the heart, gastrointestinal tract (GIT) and the brain. Once established in the aforementioned sites, the parasite triggers the activation of IO&NS pathways. For example, *T. cruzi* induces heart dysfunction via inducing inflammation, autoimmunity and O&NS. In the intestine, the parasite triggers gut microbiota changes (here presented as black bacteria) and due to increased O&NS, the epithelial lining of the intestine is loosened, allowing gram-positive bacteria/LPS to translocate to the mesenteric lymph nodes (MLNs) or the bloodstream, where they activate toll-like receptor 2 (TLR2) or TLR4, which further triggers the activation of IO&NS pathways. In the brain, IL-17 and IL-22 cause increased blood-brain barrier (BBB) permeability, allowing cytokines to reach the brain more easily. Furthermore, *T. cruzi* induces astrocytes to release PICs, leading to activation of microglia and IDO, further activating IO&NS and neuroinflammation pathways in the brain. Consequently, increased IO&NS and Glutamate (Glu), excitotoxicity and cell death are observed. All together, these changes in different organs may contribute to the depressive symptoms observed in both Chagas disease and MDD.

Finally, Chagas disease has been traditionally studied through an immunological and epidemiological viewpoint. The data discussed herein point to a matter that is currently underexplored: the neuropsychiatric aspect of the disease. Here we have reviewed that CD and depression have multiple shared IO&NS pathways that may help in the understanding of how depression arises in CD (Table 2, Fig. 1). Taking this into account may help in the design of new therapeutic strategies to be applied for the management of Chagas disease.

**Table 2:** Similarities of IO&NS pathways underlying CD and depression.

Pathways	CD	Depression
Increased levels of PICs	✓	✓
CMI activation	✓	✓
IDO activation and production of TRYCATs	✓	✓
Heart dysfunction	✓	✓
Raised levels of O&NS	✓	✓
Reduced antioxidant capacity	✓	✓
Gut microbiota changes	✓	✓
Mitochondrial dysfunction	✓	✓
Autoimmunity	✓	✓
